# Epigenetic regulation of microglia and neurons by proinflammatory signaling following adolescent intermittent ethanol (AIE) exposure and in human AUD

**DOI:** 10.3389/adar.2024.12094

**Published:** 2024-03-08

**Authors:** Fulton T. Crews, Victoria Macht, Ryan P. Vetreno

**Affiliations:** Departments of Pharmacology and Psychiatry, Bowles Center for Alcohol Studies, School of Medicine, University of North Carolina at Chapel Hill, Chapel Hill, NC, United States

**Keywords:** epigenetics, HMGB1, ethanol, alcohol, neurogenesis

## Abstract

Adolescent alcohol drinking is linked to high rates of adult alcohol problems and alcohol use disorder (AUD). The Neurobiology of Alcohol Drinking in Adulthood (NADIA) consortium adolescent intermittent ethanol (AIE) models adolescent binge drinking, followed by abstinent maturation to adulthood to determine the persistent AIE changes in neurobiology and behavior. AIE increases adult alcohol drinking and preference, increases anxiety and reward seeking, and disrupts sleep and cognition, all risks for AUD. In addition, AIE induces changes in neuroimmune gene expression in neurons and glia that alter neurocircuitry and behavior. HMGB1 is a unique neuroimmune signal released from neurons and glia by ethanol that activates multiple proinflammatory receptors, including Toll-like receptors (TLRs), that spread proinflammatory gene induction. HMGB1 expression is increased by AIE in rat brain and in post-mortem human AUD brain, where it correlates with lifetime alcohol consumption. HMGB1 activation of TLR increase TLR expression. Human AUD brain and rat brain following AIE show increases in multiple TLRs. Brain regional differences in neurotransmitters and cell types impact ethanol responses and neuroimmune gene induction. Microglia are monocyte-like cells that provide trophic and synaptic functions, that ethanol proinflammatory signals sensitize or “prime” during repeated drinking cycles, impacting neurocircuitry. Neurocircuits are differently impacted dependent upon neuronal-glial signaling. Acetylcholine is an anti-inflammatory neurotransmitter. AIE increases HMGB1-TLR4 signaling in forebrain, reducing cholinergic neurons by silencing multiple cholinergic defining genes through upregulation of RE-1 silencing factor (REST), a transcription inhibitor known to regulate neuronal differentiation. HMGB1 REST induction reduces cholinergic neurons in basal forebrain and cholinergic innervation of hippocampus. Adult brain hippocampal neurogenesis is regulated by a neurogenic niche formed from multiple cells. *In vivo* AIE and *in vitro* studies find ethanol increases HMGB1-TLR4 signaling and other proinflammatory signaling as well as reducing trophic factors, NGF, and BDNF, coincident with loss of the cholinergic synapse marker vChAT. These changes in gene expression-transcriptomes result in reduced adult neurogenesis. Excitingly, HMGB1 antagonists, anti-inflammatories, and epigenetic modifiers like histone deacetylase inhibitors restore trophic the neurogenesis. These findings suggest anti-inflammatory and epigenetic drugs should be considered for AUD therapy and may provide long-lasting reversal of psychopathology.

## Introduction

Individuals who begin drinking in their early teen years and during puberty have very high rates of adult alcohol problems and alcohol use disorder (AUD) [1]. However, causally relating early adolescent human adolescent drinking to high rates of adult AUD is confounded by multiple environmental and genetic factors that impact adolescent development, peer and family influences as well as emerging personality disorders and progressive increases in drinking trajectories into adulthood. Preclinical studies in rodents allow hypothesis testing on the impact of exposure to alcohol during adolescence that control for genetics and environment and can limit exposure to adolescent ages (i.e., without continuous alcohol exposure into adulthood). This design allows selective determination of the impact of ethanol on adolescent brain that persists into adulthood. The Neurobiology of Alcohol Drinking in Adulthood (NADIA) consortium designed the adolescent intermittent ethanol (AIE) exposure rat model to fit patterns of underage binge drinking. AIE involves alcohol exposure across what is equivalent to the teenage years in humans; in rats, this is approximately postnatal day 25 (P25) to P55, with females having puberty a bit before males, similar to humans. Following AIE, rats are allowed to mature to adulthood, usually P80–P90, equivalent to 30- to 40-year old humans, without any further alcohol exposure. The AIE model tests the hypothesis that AIE causes long-lasting persistent changes in adults that increase risks of adult alcohol problems and AUD. This model tests the impact of adolescent drinking while avoiding the human confounds, particularly genetic inheritance, that complicate understanding the strong relationship of adolescent drinking and later life AUD. In males, multiple AIE studies find increases in adult alcohol drinking [2–11]. AIE-induced adult rat drinking is increased after adolescent ethanol exposure in adults of both sexes, with females drinking more than males [12]. AIE ethanol self-administration and AIE ethanol vapor exposure also promote increases in adult operant responding for ethanol self-administration and reduce extinction [4, 13]. AIE ethanol exposure without adult ethanol exposure also increases adult anxiety and reduces behavioral flexibility and responses to acute alcohol, consistent with widespread changes in multiple cognitive-behavioral domains. Learning studies find AIE does not change young adult learning ability [4, 14–17], although complex operant tasks with rule changes and set-shifting show deficits [4] and as does some spatial–temporal object recognition [18]. Studies using the Morris water maze and the Barnes maze find initial learning is intact and not altered, but reversal learning, a measure of behavioral flexibility assessed by changing the goal location, reveals reversal deficits [18–23] due to perseveration and loss of executive function [24]. Adult rat responses in a probability discounting task that changes the ratio of arm pressing to food pellet reward find AIE increases risky choices [14, 25, 26] and enhances reward seeking in adulthood [27–30]. Another effect of AIE is heightened social anxiety in adulthood [31], particularly in males [32–34]. AIE also increases adult anxiety-like behavior using the elevated-plus maze [6, 35–37] or the light–dark box [5, 6, 37–39] or the marble-burying test [5], as well as the open-field test [21, 40]. These findings are consistent with the finding that AIE increases amygdala CRF [14]. Other reviews provide more details on the impact of AIE on persistent changes in adults behavior [24, 31, 41–44] as well as the review specifically on the role of sex in AIE [45]. In summary, adolescent alcohol exposure as modeled by AIE causes changes that increase risk factors for AUD that persist long after adolescence without additional alcohol exposure in adulthood. The mechanisms of these persistent AIE-induced changes could explain the link between age of drinking onset, lifetime AUD and alcohol-related problems.

The long-lasting changes in adult mood, cognition and reward following AIE are likely related to changes in neuronal networks that underlie self-reflection, attention and self-control mechanisms developing during adolescence. Understanding cellular mechanisms involved in adolescent maturation of brain neuronal networks and the impact of binge drinking provides important information for prevention efforts as well as targets for treatment and diagnosis. Both human [46–48] and preclinical studies [1, 24, 49] have found adolescent maturation alters brain physiology, networks, structure and function. Chronic adult binge drinking models, as well as the adolescent intermittent binge models, find changes in gene expression. Adolescent sensitivity to alcohol induced long-lasting changes in adults without further alcohol exposure in the NADIA AIE model [24, 49] in general are exaggerated responses occurring with less alcohol exposure than is needed in adult models. Proinflammatory neuroimmune genes are generally increased across models as well as in post-mortem brain of individuals with AUD. Proinflammatory genes have been linked to AUD. Transcriptome studies find changes in large numbers of gene classes that consistently include neuroimmune and epigenetic modiflying genes. More recent transcriptome studies have established the importance of single cell studies that allow links to cell and network function. Emerging studies have identified neuroimmune triggered epigenetic modifications in microglia, astrocytes, and neurons that impact neuronal networks related to mood, cognition, and salience. Epigenetic changes are reversible, providing opportunities for new therapies. However, all cells respond to their surrounding cells in different limbic and cortical brain regions that likely contribute to variation. This review will touch on epigenetic mechanisms in response to neuroimmune signaling. It introduces a complex cytokine-like molecule, high-mobility group box 1 (HMGB1), as a key brain proinflammatory signal linked to alcohol-induced changes. Microglia are the innate immune cells of brain and are primed or sensitized by alcohol-linked HMGB1 proinflammatory signals. Microglial and astrocyte changes during cycles of alcohol exposure are proposed to interact with neurons through signals altering gene expression through complex mechanisms. AIE-induced changes in cholinergic (ChAT) basal forebrain neurons and hippocampal dentate gyrus neurogenesis are reviewed as examples of how neuronal networks linked to cholinergic arousal and new neuron formation undergo persistent adult cognitive deficits that can be restored through reversal of proinflammatory-epigenetic signaling.

## Epigenetic mechanisms of AIE-induced AUD-like pathology

The mechanisms of AIE-induced changes in adult rat brain are linked to increases in neuroimmune gene expression across neurons, microglia, astrocytes and likely other brain cell types. Epigenetics has emerged as a mechanism of persistent, long-lasting changes in gene expression in response to environment, including enriched, stressful or trauma-induced changes [36, 49, 50]. Epigenetic gene regulation includes histone and DNA methylation and microRNA regulators of gene expression and cell phenotype reprogramming that have emerged as mechanisms of alcohol-induced changes in brain that are linked to proinflammatory signaling. Epigenetics shifts transcription through silencing or enhancing gene transcription [51–53]. Although neurons connect across brain regions, glial-neuronal signals regulate synapses and other interactions within each brain region. Studies of AIE find reduced trophic factor expression with increased proinflammatory gene expression which are persistent shifts in cellular transcriptomes lasting to adulthood, and which are reversible with anti-inflammatory or epigenetic modifying drugs. Binge alcohol exposure was first discovered to induce long-lasting changes in brain neuroimmune gene expression [54–57]. Chronic ethanol exposure of mice was discovered to increase brain Toll-Like receptors (TLR) and sensitize brain TLR4 [58] and TLR3 proinflammatory responses [59] that has emerged as mechanism regulating alcohol self-administration and preference in mice [60, 61], as well as following AIE in rats [24, 49]. Cycles of alcohol-induced innate immune memory processes increase TLR expression in brain, priming microglia and other cells and thereby increasing proinflammatory responses [62–64]. There are a large number of genes associated with the immune system, including adaptive immunity T and B cell lymphocytes, as well as innate immunity tissue-specific and blood monocytes [65]. Healthy brain does not have T or B lymphocytes or their associated antibodies and there are low levels of expression of innate immune genes with some being expressed transiently in neurons during development or initiation of synaptic plasticity. A large number of studies currently link ethanol drinking and preference to neuroimmune signaling using transcriptomic models [66–68], transgenic animal models [69, 70], post-mortem human brain immunohistochemistry and PCR [71–74], and AUD models [75]. In general, brain neuroimmune gene expression refers to genes associated with innate immune signaling, particularly proinflammatory cytokines such as TNFα, IL1β, and IL6. In healthy brain, these genes are expressed at very low levels but are sensitive to drugs, stress, and other environmental factors. A characteristic of proinflammatory innate immune signaling is that an initial signal from one cell activates multiple other cells and itself to increase expression of multiple proinflammatory cytokines, chemokines, and other genes. This results in many proinflammatory signaling molecules being involved in the lasting changes induced by chronic ethanol exposure. This review will focus on HMGB1, an endogenous protein expressed in all brain cells that has both nuclear and immune signaling proinflammatory functions [76]. High-mobility group (HMG) proteins were first identified as a class of nonhistone proteins that contribute to packaging DNA into chromosomes, with high-mobility group box 1 (HMGB1), emerging as an actively released protein with a key role in immune signaling [76, 77]. HMGB1 was discovered to bind neuroblasts and called amphoterin, but has emerged as an endogenous cytokine-like molecule that can activate multiple TLRs, previously discovered to respond to complex bacterial products in the immune system, but rarely studied in sterile brain. Examples of AIE-altered HMGB1 signaling and persistent changes in adult brain include adult hippocampal neurogenesis, microglial priming, and loss of basal forebrain cholinergic neurons. The mechanisms of AIE-induced persistent changes in HMGB1 and neuroimmune signaling are linked to lasting changes in adult perseveration, cognition, and AUD risk behaviors. (See Table 1).

**TABLE 1:** Select articles on HMGB1, adolescence, and alcohol.

Preclinical and clinical alcohol exposure effects on HMGB1 primary literature				
Species	Exposure	Assessment	Results	Reference
Rat (Wistar)	AIE	Prefrontal cortex (PL, IL)	↑ HMGB1 (IHC, mRNA), also TLR4, TLR3 (mRNA) in P56 and P80 adult rats. HMGB1 colocalizes with neurons (NeuN). AIE rats also exhibit reversal learning deficits.	[23]
Human	AUD	Orbitofrontal Cortex	↑ HMGB1 correlated with earlier age of drinking onset (IHC), also ↑ RAGE	[74]
Rat (Wistar)	AIE	Orbitofrontal Cortex	↑ HMGB1 (IHC) and ↑RAGE	[74]
Rat (Sprague)	CE (7% liquid diet, 15 days), or CIE (7% liquid diet intermittent)	Cortex (whole brain)	↑ HMGB1 (mRNA) during CE and CIE withdrawal but not intoxication; also increased TLR4 (mRNA) but no change in MyD88 (mRNA) or NFĸB (mRNA)^a^ ↑ HMGB1 (mRNA) during CE and CIE withdrawal blocked by CRF1 antagonist (CP154,526: 10 mg/kg) and ethyl pyruvate (75 mg/kg) but not the HMGB1 antagonist glycyrrhizin	[78]
Human	AUD	Orbitofrontal Cortex	↑ HMGB1 correlates with TLR and age of drinking onset	[72]
Rat (Wistar)	0 → 100 mM EtOH	hippocampal- entorhinal cortex organotypic slice culture	↑ HMGB1 (mRNA), ↑ HMGB1 released into media (ELISA)	[72]
Rat (Wistar)	0 → 100 mM EtOH	hippocampal- entorhinal cortex organotypic slice culture	Ethanol dose dependently ↑ HMGB1 (mRNA) and ↑ HMGB1 released into media (ELISA). Acetyl-HMGB1 is released; HDAC inhibitors also increase acetyl- HMGB1 release into media	[79]
Rat (Wistar)	AIE	Hippocampus	↑ HMGB1 (mRNA)	[80]
Human	AUD	Hippocampus	↑ HMGB1 (WB) ↑ HMGB1/1L-1β complexes (WB)	[81]
Mouse	Acute 6 g/kg i.g.	Whole brain Cortex Plasma Liver	↑ HMGB1 (ELISA, IHC, WB) ↑ HMGB1/1L-1β complexes (Western blot, IHC) ↑ HMGB1 (ELISA) ↑ HMGB1 (WB)	[81]
Human	AUD	Hippocampus	↑ HMGB1 in Human AUD Hippocampus (ELISA)	[82]
Rat	25–100 mM ethanol (48 h)	hippocampal- entorhinal cortex organotypic slice culture	↑ MV-HMGB1 (ELISA) and miRNA Let7 ↑ HMGB1/Let7 complexes in MV (ELISA)	[82]
Rat (Wistar)	AIE	Hippocampus	↑ HMGB1, TLR4, TNFα, IkBα (mRNA) and loss of neurogenesis (DCX, IHC) ^a^Prevented with concurrent voluntary exercise or indomethacin	[83]
Human (young adult) ♀ ♂	Binge Drinkers	Serum	↑ HMGB1 (ELISA) in female but not male subjects following acute binge alcohol	[84]
Rat (Wistar)	AIE	Hippocampus	↑ HMGB1 (IHC), ↑ RAGE, ↑ TNFRSF25, cleaved caspase-3, pNFĸB-p65 ^a^HMGB1 changes not reversed with donepezil; other proinflammatory markers reversed by donepezil	[85]
Mouse/Human cell line	100 mM EtOH (24 h)	BV2, SH-SY5Y BV2+ SH-SY5Y co-culture	24 h EtOH did not impact HMGB1 (mRNA) in BV2, SH-SY5Y or co-culture 24 h EtOH ↑HMGB1 release into media in BV2 and SH-SY5Y cultures but not in co-cultured BV2+SH-SY5Y preps. IL-4 and IL13 mRNA increased in co-culture EtOH EtOH ↑ TLR4 (mRNA)in co-culture BV2/SH-SY5Y, but co-culture attenuated EtOH TLR3/TLR7 (mRNA) and iNOS (mRNA)	[86]
Human (AUD)	AUD	Orbitofrontal Cortex	AUD increases multiple TLR and NFĸB genes that correlate with increased expression of HMGB1	[73]
Rat (Wistar)	AIE	Basal Forebrain	↑ HMGB1 (IHC) with ↑TLR4, ↑ pNFĸB p65, and ↑ RAGE as well as ↑ H3K9me2 and decreased ChAT by AIE^a^ Galantamine prevented/reversed AIE-induced changes in adulthood	[87]
Rat (Wistar)	AIE	Dentate gyrus of the hippocampus	↑ HMGB1 (IHC) and other proinflammatory markers including CCL2, COX2 and cleaved Caspase-3 while decreasing neurogenesis (DCX) ^a^galantamine prevented/reversed	[88]
Human ♀ ♂	AUD, ALD	Serum	↑ HMGB1 in ALD relative to AUD (ELISA); predicts mortality in AUD.	[89]
Rat (Wistar)	*In vivo*: AIE *Ex vivo*: dsHMGB1 and rHMGB1, 100 mM EtOH for 4 days	*In vivo*: Basal Forebrain *Ex vivo*: BFCN organotypic slice culture	*In vivo*: ↑ HMGB1 (mRNA) *Ex vivo*: dsHMGB1 and rHMGB1 both reduce ChAT. Ethanol releases HMGB1 into media. REST and G9a induction lead to ChAT gene silencing. Loss of ChAT blocked by HMGB1 antagonist glycyrrhizin	[90]
Rat (Wistar) ♀ ♂	AIE	Dentate gyrus of the hippocampus	↑ HMGB1 (IHC) ^a^Indomethacin reversed AIE-induced loss of neurogenesis and cholinergic markers and reduced HMGB1 (IHC)	[91]

Other HMGB1-RELATED primary literature				
Species	Exposure	Assessment	Results	References
Rat (unspecified)	0–5 mM Glutamate; 0–100 µM NMDA	hippocampal- entorhinal cortex organotypic slice culture	Glutamate dose-dependently ↑ HMGB1 release into media parallel to ↑ cell death (exclusion dye propidium iodide). NMDA similarly dose-dependently ↑ HMGB1 release into media parallel to ↑ cell death (exclusion dye propidium iodide).	[92]
Vglut2-Cre/ChR2-eYFP mice	ChR2 stimulated	*In vivo*: DRG *Ex vivo*: DRG neuronal culture	↑ HMGB1 cytoplasmic translocation (IHC) ↑ HMGB1 release (WB/ELISA)	[93]
Syn-Cre/HMGB1fl/flMice	Neuronal HMGB1 ablation	DRG	Neuronal HMGB1 ablation reduced hyperalgesia following sciatic nerve injury and attenuated proinflammatory cytokine and chemokine responses (ELISA: TNFα, CXCL1, IL18)	[93]
Rat (Wistar)	*In vivo*: LPS (1 mg/kg, i.p.); *Ex vivo*: LPS (100 ng/mL), dsHMGB1	Basal forebrain (*in vivo*); BFCN organotypic slice culture (*ex vivo*)	dsHMGB1 and LPS trigger TLR4 induction of REST and G9a gene silencing to cholinergic transcriptome.	[94]
CD-1 mice	Radioactive labeled HMGB1	Whole brain Serum	HMGB1 is transported across the BBB in both directions. LPS exposure ↑HMGB1 transport in part by disrupting the BBB and in part through a transport mechanism.	[95]
Swiss albino or transgenic Thy1-ChR2- YFP and hGFAP-GFP adult mice	optogenetic stimulation or pinprick for cortical spreading depolarization	Cortex	↑ HMGB1 nuclear translocation (IHC) and ↑ HMGB1 extracellular vesicles with some indication of astrocyte-HMGB1 but not microglial-HMGB1 interactions	[96]

HMGB1-Related review articles	
Findings	Reference
This review covers the rapid release of HMGB1 from neurons during a seizure, increasing astrocyte and microglial IL-1β/HMGB1 synthesis and release. Long lasting decreases in seizure threshold are linked to persistent increases in these signals.	[97]
Proposed the hypothesis that neuroimmune signaling contributes to the neurobiology of alcohol and substance use disorders.	[55]
The review covers evidence supporting drug induced increases in TLR in brain, particularly microglia, that respond to HMGB1 and microRNAs (miRNAs). Studies supporting ethanol enhanced TLR innate immune signaling changes gene transcription through epigenetic mech anisms alternating synapses and neuronal networks. Addiction involves progressive stages of drug binge intoxication and withdrawal that are linked to progressive increases in TLR signaling.	[71]
This review discusses HMGB1 oxidation-reduction and changes activities through multiple cell surface receptors. Also, this review discusses recent discoveries indicating that HMGB1 released from neurons mediates inflammation via the TLR4 receptor system.	[98]
The studies reviewed support roles for neuroimmune signaling as well as epigenetic reprogramming of neurons and glia, which create a vulnerable neuro- environment. Some of these changes are reversible, giving hope for future treatments to prevent many of the long-term consequences of adolescent alcohol abuse.	[99]
AIE increases adult alcohol drinking, risky decision-making, reward-seeking, and anxiety as well as reducing executive function that increase risks for AUD. AIE causes persistent increases in adult brain neuroimmune signaling high-mobility group box 1 (HMGB1), TLR, RAGE and other innate immune genes. These genes are also increased in human AUD brain. HMGB1 release by ethanol, both free and within extracellular vesicles shifts transcription and cellular phenotype. For example, RE-1 silencing transcript blunts cholinergic gene expression, shifting neuronal phenotype. Inhibition of HMGB1 neuroimmune signaling, histone methylation enzymes, and galantamine, the cholinesterase inhibitor, both prevent and reverse AIE pathology. These findings provide new targets that may reverse AUD neuropathology as well as other brain diseases linked to neuroimmune signaling.	[1]
This is a review of HMGB1 immune cell functions including promoting DNA damage repair in the nucleus, sensing nucleic acids and inducing innate immune responses and autophagy and stimulating immunoreceptors. Signaling, cellular functions and clinical relevance of HMGB1 in various diseases are discussed.	[77]

^a^ AIE, adolescent intermittent ethanol; AUD, alcohol use disorder; ALD, Alcohol-related Liver Disease; BBB, blood brain barrier; CCL2, c-c motif ligand 2; COX2, cyclooxygenase-2; DCX, doublecortin; DRG, dorsal root ganglion; dsHMGB1, disulfide high mobility group box 1; ELISA, enzyme-linked immunosorbent assay; EtOH, ethanol; IHC, immunohistochemistry; IL, infralimbic; NeuN, neuronal nuclear protein; LPS, lipopolysaccharide; MV, microvesicle; pNFĸB, phosphorylated nuclear factor kappa-light chain enhancer of activated B cells; PL, prelimbic; RAGE, receptor for advanced glycation end products; TNFRSF25, tumor necrosis factor receptor superfamily 25; WB, western blot.

**FIGURE 1:**
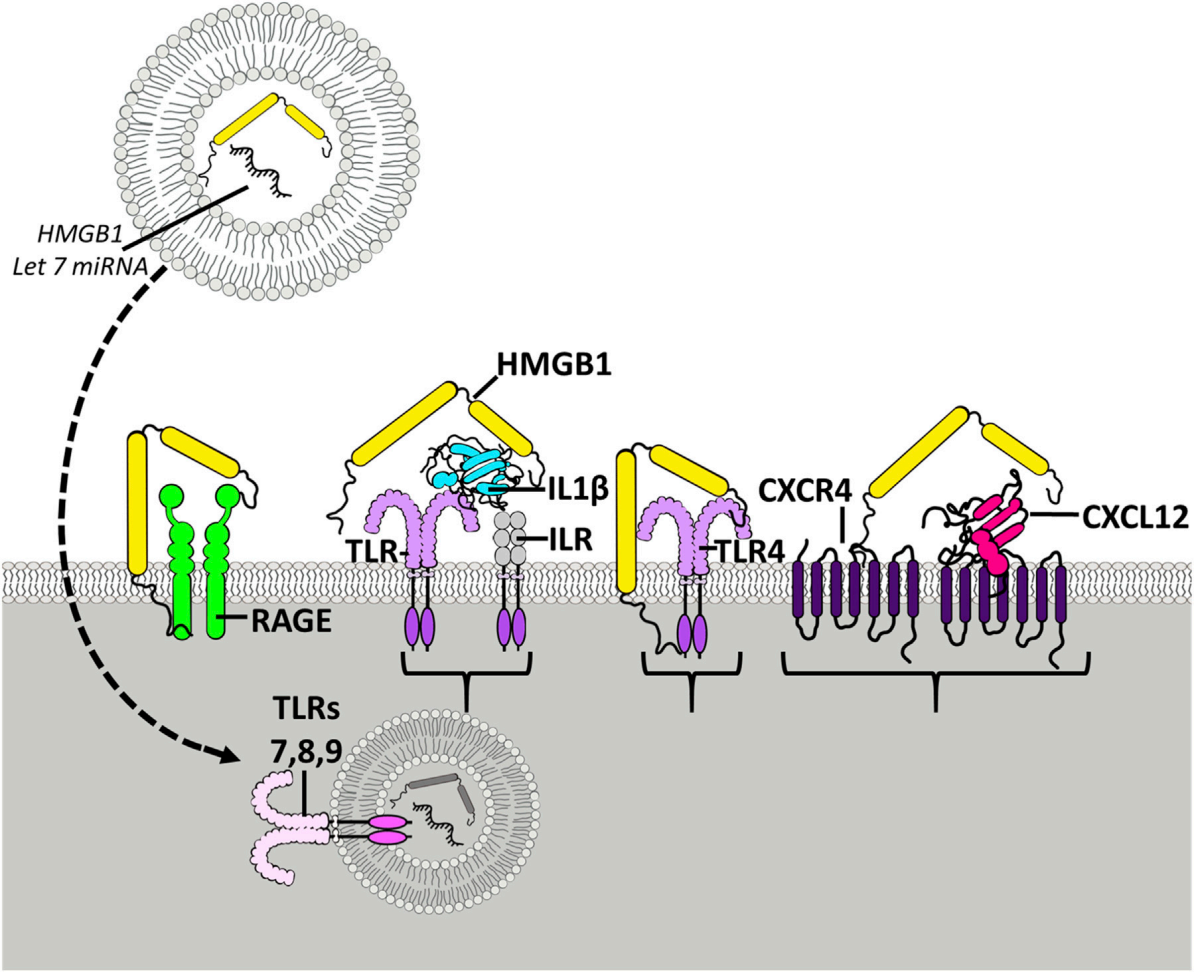
HMGB1 activates multiple receptors spreading neuroimmune signaling. Shown is the HMGB1 (yellow) molecule with two yellow Box sections, known as Box A and Box B, that bind to different molecules and receptors. The Box sections aggregate-stabilize (dimerize) receptor subunits, increasing activation. HMGB1 can stimulate TLR4 receptors directly and as heteromers with other TLR agonists. TLR receptors are members of the TLR-IL1 receptor family that are activated by agonist dimerization. Receptors are drawn as active dimers with HMGB1 bridging dimers, the hypothetical mechanism of HMGB1 potentiating receptor responses. Shown is HMGB1 alone stimulating TLR4 and RAGE receptors. HMGB1 is known as a “sticky” protein binding to lipids, RNA, DNA, and chemokine proteins. On the right is shown HMGB1-CXCL12 heteromers bridging G-protein receptors. HMGB1 has been found to enhance the potency of CXCL12 at CXCR4 receptors, G-protein-linked chemokine receptors [105] activated by dimerization. Another example involves IL1β-ILR receptors (TLR/ILR receptor family) which act through HMGB1/IL1β heteromers, increasing potency at the ILR over that of IL1β alone [100]. Similarly, studies find microglial activation releases HMGB1 as a heteromer in microvesicles with microRNA let-7, an endogenous TLR7 agonist that when combined with HMGB1, that is able to activate TLR7 in adjacent neurons [106]. HMGB1 complexes can activate essentially all TLRs [102], contributing to HMGB1 as a proinflammatory signal. HMGB1 has broad neuroimmune stimulating activity crossing multiple innate immune receptors.

HMGB1 expression in increased in post-mortem human AUD hippocampus as well as ethanol-exposed rats and mice and AIE-treated adult rats [71, 100]. AIE also induces subtle but persistent increases in hippocampal expression of the proinflammatory signaling factors chemokine C-C motif ligand 2 (CCL2), cytokines TNFα and IL1β, and cyclooxygenase-2 as well as expression of innate immune signaling Toll-like receptors (i.e., TLR1, TLR2, TLR4, TLR5, TLR6, TLR7, and TLR8) [73] and the receptor for advanced glycation end products (RAGE) and other proinflammatory signaling cytokines signal through feed-forward amplification innate immune receptors and their activating ligands. Interestingly, HMGB1 is actively released following acetylation [101] and we found ethanol, histone deacetylase inhibitors, and glutamate increase hippocampal brain slice culture release of HMGB1 into the media [79]. Studies in culture find ethanol releases HMGB1 from neurons [79] and microglia [82]. HMGB1 can form monomers as well as dimers and heteromeric complexes that function as a pan-proinflammatory amplifying factor. HMGB1 heteromeric complexes form with cytokines, extracellular DNA, RNA, and damage-associated molecular pattern (DAMP) molecules [93]. HMGB1 heterocomplexes are able to activate TLRs, making TLRs an important proinflammatory signal [102]. For example, TLR7 is activated by RNA, including endogenous miRNA let7 and HMGB1-let7 dimers, which are both potent agonists. Interestingly, ethanol releases HMGB1-let7 dimers in extracellular vesicles (EVs) from microglia, triggering TLR7-mediated pathology [82]. Multiple studies suggest TLR7 is linked to increases in preclinical alcohol drinking and preference [103, 104]. The ability of HMGB1 to activate and amplify proinflammatory signals positions it as a key target to block proinflammatory gene induction (Figure 1). Alcohol and substance abuse disorders involve a progression of increased drug taking with activation of reward centers, followed by mood dysfunction and limbic involvement with increasing involvement of prefrontal and other cortical dysfunction [107] that could represent progressive increases in HMGB1 and/or other neuroimmune signals. It is nearly impossible to measure all proinflammatory signals, with most studies focusing on TNF, IL1B, IL6 or CCL2. The classical acute phase innate immune systemic blood response to infection involves these and other proinflammatory cytokines and chemokines, consistent with all being representative of neuroinflammation. This is an oversimplification since neurons, astrocytes, microglia and other brain cells respond to an initial proinflammatory response with different cytokines that vary dependent upon the surrounding mileu and brain region that alters the spread of proinflammatory signaling. One example linking HMGB1, proinflammatory signaling and ethanol pathology is sensitization to pain. Pain as assessed by tactile allodynia increases following nerve injury due to changes in neurons and the local microglia [108]. Spinal cord microglia are activated contributing to pain sensitization [108]. In models of pain, nociception sensory neurons have increased HMGB1 and increases in HMGB1 release with increased pain. Antibodies to HMGB1 have been block neuropathic pain [109]. Neuronal activation using optogenetic mechanisms increases release of HMGB1 [93] that can activate microglia. Further, silencing of HMGB1 protects against both nerve injury and proinflammatory pain models [93]. These findings are consistent with ethanol induction and neuronal release of HMGB1 contributing to local microglial sensitization that persists and amplifies proinflammatory responses that impact neurocircuitry. Studies finding HMGB1 release from multiple brain cells is consistent with initiating proinflammatory signaling, although the details on reward, affect, and cognitive neurocircuitry is not known. Understanding the mechanisms of progressive increases in brain HMGB1-TLR proinflammatory signaling across brain regions, neurocircuits, and components of psychopathology will benefit both prevention and treatment efforts.

## Microglia, HMGB1, and alcohol

Microglia are brain-specific monocyte-like cells that are long-lived but can also divide from endogenous progenitors throughout the lifespan [110]. Microglia within each brain region is relatively stable and if altered, microglia proliferate to return to the “homeostatic” density, suggesting local regulatory microglial niche mechanisms [111]. Microglia are suggested to control the escalation of drinking in mouse models of alcohol dependence [112], consistent with escalation of drinking being linked to amplification of HMGB1 -proinflammatory signaling increases with repeated exposure [71, 100]. During striatal development microglia regulate dopamine receptors, with male sex-specific microglial elimination of striatal synaptic dopamine D1 receptors through microglial-transcytosis, i.e., synaptic receptor specific phagocytosis, that precedes the development of male specific adolescent play behaviors [113]. In transgenic mice with depleted numbers of microglia, there is reduced adolescent synaptic pruning, resulting in more synapses but reduced cortical function [114]. Interestingly, cortical microglial gene expression correlates with cortical thickness during childhood and early adolescence [115], and cortical thickness is linked to development of adult characteristics [116, 117]. Though microglia are critical for neurodevelopment during adolescence, in general, little is known about microglia and their role in specific neurocircuitry. What is known is that microglia have multiple phenotypes that are regulated through epigenetic mechanisms [118] and adolescent ethanol exposure causes long lasting sensitization and other alterations in brain microglia [49, 119, 120].

Microglia contribute to acute alcohol responses [82, 121, 122] and become sensitized to proinflammatory signals like HMGB1. Sensitization or priming of microglia by stressors or TLR agonists persists [123, 124], and priming increases expression of complement pathways that regulate synaptic plasticity [125]. For example, AIE adolescent binge ethanol exposure followed by 45 days of abstinence increases adut restraint stress Cd11b+ microglia activation in frontal cortex and amygdala [121]. Adolescent stress also increases adult microglia responses to lipopolysaccharide (LPS) [126], consistent with studies finding ethanol sensitizes to LPS [127]. Another adolescent binge ethanol exposure study found disruption of novel object learning and hippocampal long-term synaptic depression are blocked by microglial inhibitor minocycline and TLR4 antagonist TAK-242, as well as the anti-inflammatory drug indomethacin [128]. Another AIE study found increased pain sensitivity in adults that was alleviated by minocycline [129]. These studies support AIE priming of microglia, although stress can also prime microglia; adolescent alcohol and stress sensitize and synergize to increase proinflammatory responses in some brain regions but not others [121]. Recent studies report blood monocytes of individuals with AUD are primed to TLR4 proinflammatory responses [130]. These studies suggest microglial priming contributes to increases in alcohol drinking and AUD psychopathology.

## Immune signaling and acetylcholine

Although in general microglia and proinflammatory signaling are linked to the mechanisms that underlie the development of AUD, proinflammatory responses are complex. One example is the pain circuit, which has both central and peripheral components and the anti-inflammatory actions of acetylcholine [131, 132]. Both adult and adolescent AIE are found to sensitize pain responses [133, 134]. HMGB1, microglia and proinflammatory signals are linked to pain sensitivity. Acetylcholine inhibits microglia and the vagus nerve sends projections to the organs that inhibit proinflammatory responses with acetylcholine [135–137]. The inflammatory reflex signals are anti-inflammatory nerve signals that stimulate a subset of immune cells to secrete acetylcholine, which interacts with alpha 7 nicotinic acetylcholine receptors to inhibit proinflammatory mediators [138, 139]. Thus, acetylcholine is known to reduce proinflammatory signaling and brain regions with high levels of acetylcholine will show less proinflammatory induction by ethanol and other insults than brain regions without any cholinergic anti-inflammatory signaling.

## HMGB1 and epigenetic regulation of forebrain cholinergic neurons

Forebrain cholinergic neurons projection to multiple cortical and limbic brain regions, including the cortex, hippocampus, and amygdala. Cholinergic neurons modulate arousal, cognitive and emotion [140, 141]. AIE reduces expression neuronal choline acetyltransferase (ChAT) in the medial basal forebrain and shrinks remaining ChAT + IR cholinergic neurons size [18, 20, 22, 40, 142–145]. The vesicular ACh transporter (VAChT), and the high- and low-affinity nerve growth factor receptors TrkA and NGFR, all cholinergic neuron markers are also decreased [22, 83, 142]. The AIE-induced loss of basal forebrain cholinergic neurons is accompanied by diminished ACh prefrontal cortical efflux during maze performance [144]. The forebrain ChAT+ cell loss is selective, since parvalbumin GABAergic neurons in the basal forebrain are not reduced by AIE [20]. AIE deficits in reversal learning are linked to the ChAt loss by anti-inflammatory indomethacin, exercise, and galantamine treatments during AIE that prevent the loss of ChAT+ neurons and cognitive deficits [22, 142, 145]. The TLR4 agonist lipopolysaccharide (LPS) activates brain proinflammatory signaling and treatment during adolescence mimics the AIE-induced loss of ChAT [40, 145]. AIE induces forebrain TLR4 and RAGE receptors, HMGB1, and the nuclear transcription factor pNFkB p65 proinflammatory signaling transcription factor [40, 145]. Rat voluntary wheel running exercise, and indomethacin prevent AIE induction of HMGB1-TLR4/RAGE-pNFκB p65+IR within ChAT + IR neurons, their loss and shrinkage [145]. Historically, loss of terminally differentiated ChAT+ neurons was interpreted as cell death and considered irreversible. However, emerging studies find brain proinflammatory signals are induced by epigenetic changes in microglia and neurons that are reversible. Studies found that reduced ChAT+ neurons, and shrunken ChAT+ neurons could be restored after AIE treatment. Exercise running wheels reversed AIE increased forebrain HMGB1-TLR4 and RAGE-as well as the loss of ChAT+, TrkA+, and NGFR+ cholinergic neurons and somal shrinkage. There were no changes in total NeuN+ neuron numbers and no neurogenesis, suggesting neurons did not die but only lost the cholinergic phenotype, allowing restoration [22, 142]. These findings were extended with anti-inflammatory treatments indomethacin and galantamine, which acts through enhanced acetylcholine as an anti-inflammatory treatment. More recent studies have discovered transcriptional repressor RE1-silencing transcript (REST; also known as neuron-restrictive silencer factor [NRSF]) [146, 147] regulate cholinergic gene expression [147] and is known to bind methyltransferase G9a, increasing histone H3K9 dimethylation that represses gene expression [148, 149]. HMGB1 signaling was discovered to increase REST-G9a silencing of multiple genes that define a cholinergic neuron, and that reversal of REST-G9a silencing restored the cholinergic neurons [90]. The findings that adolescent binge ethanol exposure and neuroimmune induction have epigenetic components that are reversible create promise for new AUD therapies [1, 52, 150–153].

## The hippocampal neurogenic niche and alcohol

The hippocampal dentate gyrus subgranular zone is a unique brain region where new neurons are formed well into adulthood. New neurons form from proliferating progenitors that become mature neurons which functionally integrate into neurocircuitry in adulthood [154, 155]. The local environment is a “neurogenic niche” regulating the birth, differentiation, and functional integration of hippocampal newborn neurons. The niche is sensitive to disruptions that alter trophic support due to increased proinflammatory signaling [156]. Ethanol exposure reduces hippocampal neurogenesis due in part to changes in the neurogenic niche [156]. Models of AUD binge drinking in adults find ethanol inhibits hippocampal neurogenesis transiently that recovers during abstinence [54, 119, 157]; however, adolescents which have about 4-fold more neurogenesis than adults [158, 159] show a persistent loss following AIE adolescent AIE exposure, far greater than that with identical adult alcohol treatment [160]. Further, the AIE-induced loss of neurogenesis persists for months, likely for life [80]. AIE inhibition of hippocampal neurogenesis following AIE is associated with adult reversal learning impairments, increased perseveration and/or loss of cognitive flexibility, which persist at least to middle age in rodents [80, 161]. The niche is disrupted by AIE. AIE increases hippocampal proinflammatory HMGB1, COX2 and other proinflammatory genes [83, 85, 88]. And reduces expression of trophic factors, specifically BDNF [37]. Interestingly, the AIE-induced loss of adult neurogenesis is reversible. Exercise and anti-inflammatory drugs (e.g., indomethacin, donepezil, and galantamine), as well as the epigenetic histone deacetylase inhibitor, trichostatin A (TSA) prevent and/or restore the AIE-induced loss of neurogenesis as well as the lasting perseveration and loss of behavioral flexibility [37, 83, 85, 88]. AIE increases HMGB1 and other proinflammatory genes [83] and decreases in the trophic factor BDNF [37], suggesting that AIE disrupts the neurogenic niche through a transcription shift increasing proinflammatory genes while reducing trophic gene expression through epigenetic gene silencing and enhancer mechanisms. The proinflammatory HMGB1 reduced trophic expression changes in gene expression and the loss of neurogenesis that are reversed by anti-inflammatory treatments like indomethacin [91] as well as the histone deacetylase inhibitor TSA [37] supports the hypothesis of epigenetic shifts driven by proinflammatory signals that reduce neurogenesis. More specifically, indomethacin, the non-steroidal anti-inflammatory drug and the cholinesterase inhibitors galantamine and donepezil reverse AIE-induced loss of neurogenesis and increases in hippocampal HMGB1 [85, 88]. TSA, a histone deacetylase inhibitor that reverses epigenetic proinflammatory activation in microglia as well as other cells, restores hippocampal BDNF and AIE-neurogenesis [37]. TSA also reverses AIE-induced changes in amygdalar histone acetylation, reverses AIE adult anxiety, and reverses AIE induced increases in ethanol self-administration [6]. Restoration of neurogenesis also restores cognitive flexibility deficits during reversal learning on the Morris water maze [83]. The changes in the niche are complex. For example, AIE reduces cholinergic innervation of the niche, and anti-inflammatory treatment restores cholinergic innervation with the return of neurogenesis (for review see [156]. Although the folklore of Alcoholics Anonymous is “Once an alcoholic, always an alcoholic,” thereby arguing AUD is a chronic disease, the findings that the AIE-induced AUD-like pathology is reversible provide a foundation for AUD cures. Understanding the brain region-specific mechanisms of AIE persistent pathology could lead to new and novel therapies for AUD.

## Discussion and summary

Adolescent drinking is known to result in high rates of adult alcohol problems and lifelong AUD. To tests hypotheses on the lasting impact of adolescent drinking, the AIE adolescent binge drinking model assesses behavior and neurobiological mechanisms after several weeks of abstinent maturation to adulthood. AIE increases alcohol drinking and preference, anxiety, reduces adult social interaction, increases pain sensitivity and other hyperkalifia-like symptoms, as well as altering decision making while increasing perseveration and reversal learning deficits. Environment and access to alcohol contribute to the development of AUD; increased alcohol drinking, hyperkalifia, and reduced executive function following AIE are consistent with increasing risks of developing AUD in adulthood. The high rates of lifetime AUD following adolescent binge drinking have been suggested to be due to a lower adolescent intoxication response to alcohol, resulting in greater and sometimes extreme binge drinking that insults the developing adolescent brain. Adolescent brain is more sensitive to acute binge alcohol exposure [162, 163]. Although brain cellular damage is increased in models of adolescent binge drinking [162] and human AUD brain is generally smaller than moderate drinking controls, AIE studies indicate that the persistent, long-lasting impact of adolescent binge drinking is far broader than cellular damage due to changes in cells and neurocircuits induced by alcohol that persist long after alcohol exposure.

**FIGURE 2:**
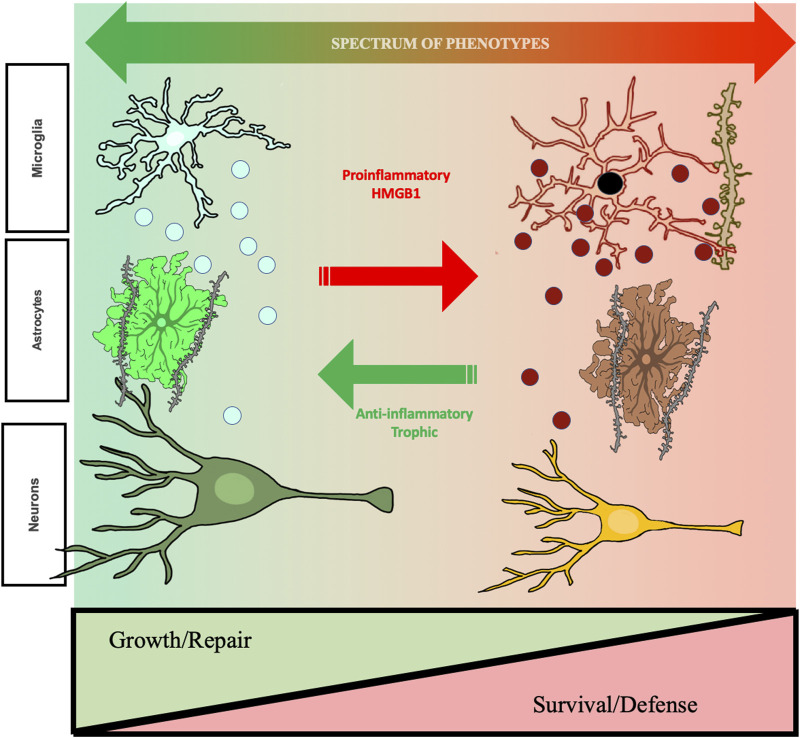
Hypothetic mechanism of ethanol-induced changes in cellular phenotypes related to changes in aud behavioral phenotypes. The studies reviewed find repeated cycles of binge drinking prime microglia, increase proinflammatory HMGB1, and alter brain and behavior that increases risk for AUD. Shown are microglia, astrocytes, and neurons. Left side green healthy microglia are trophic and release factors supporting a local growth repair milieu with other brain cell types including astrocytes, that help maintain synapses, and neurons. Chronic ethanol exposure “primes” microglia, that over repeated cycles converting them to a proinflammatory phenotype with increases in expression of CD68, a dark microglial phagocytic protein stain, and secretion of TNFα that persist for long periods and may impact synapse phagocytosis. Chronic studies of adolescent AIE find astrocytes also undergo a phenotype change, with alterations in GFAP and soma as well as reduced astrocyte-excitatory synapse PSD95 contacts. These long-lasting changes in astrocytes may represent a phenotype shift. Under healthy physiological conditions, astrocytes close synaptic contact with glutamatergic terminals where they regulate the synaptic environment and mediate glutamate homeostasis. This can be visualized using a combination of excitatory synaptic markers, glial-fibrillary actin protein (GFAP), and virus mediated astrocyte labeling with GFP. AIE causes hippocampal astrocytes to increase GFAP immunoreactivity in both sexes, indicating a shift towards a reactive phenotype, coupled with retractions of astrocytic processes from contact with excitatory synapses. These changes have critical functional implications for the role of astrocytes on mediating glutamate transmission, innate immune activation, and excitotoxicity. As described in the text, cholinergic neurons also change phenotype, some neurons lose the cholinergic phenotype and others show shrinkage of soma and loss of cholinergic markers in frontal cortex and hippocampal projections. Some neurons are no longer cholinergic, and remaining neurons have small soma suggest neuronal phenotype changes. These changes are associated with cognitive deficits, suggesting altered neurocircuitry. Evidence supports epigenetic mechanisms persistently shift cellular phenotype, but are reversible by exercise and other anti-inflammatory treatments. Reversal of phenotype changes also reverses behavioral deficits. Studies in the text support proinflammatory activation as altering cellular phenotypes from healthy growth repair to survival phenotypes that associate with ethanol induced changes in cognition and reward seeking, behavioral phenotypes with increased risks for AUD. Taken together, these results support that ethanol-induced changes in neuroimmune signaling mediate changes neurocircuitry that increase risks for AUD, but that are reversible.

The discovery that neuroimmune signaling is linked to alcohol use disorder and alcohol drinking has emerged during the past decade. This review focuses on HMGB1, a molecule that is expressed in all brain cells, is localized in the nucleus, and that is actively released from cells following acetylation by histone acetylases. Ethanol increases neuronal histone acetylation in brain [164], and in brain slice cultures, ethanol releases acetylated HMGB1 into the media. HMGB1-histochemistry shows increases in neuronal cytoplasm consistent with active neuronal release [165]. Although poorly understood and confounded by cell death-triggered release, ethanol likely releases HMGB1 from multiple brain cell types, which sensitizes microglia and astrocytes to progressive increases in a large number of proinflammatory genes. Dependent upon brain region, each acute binge drinking episode can amplify and spread proinflammatory signaling. Proinflammatory signaling is associated with sickness behaviors that fit well with the negative emotional, hyperkatifeia [166, 167] affect stages of the development of AUD. Interestingly, ethanol acutely blocks monocyte responses that change within hours, increasing proinflammatory gene expression; that is, alcohol withdrawal coincides with increases proinflammatory cytokines [54, 121]. Binge drinking and associated acute withdrawals are proposed to prime microglia, and likely astrocytes, sensitizing and amplifying proinflammatory genes. Repeated withdrawals drive hyperkatifeia responses that promote further drinking that progressively involves altered neurocircuitry across reward, negative affect-hyperkatifeia linked and finally executive control dysfunction, leading to perseverative compulsive craving. Although it is poorly understood how various neurocircuits become progressively involved in the development of AUD, some insight is provided by studies of HMGB1 and hippocampal seizures. Hippocampal seizures induce persistent increases in HMGB1-TLR4 and IL1β which increases excitability, reducing seizure thresholds, i.e., sensitizing to future seizures, due to increases in HMGB1 and IL1β [97]. Similarly, cycles of chronic intermittent ethanol that progressively increase anxiety and negative effect are linked to HMGB1 amplification of amygdala TLR4 and changes in CRF with multiple withdrawals that are blocked by CRF1A and HMGB1 antagonists [78]. Although adolescents are proposed to be more sensitive to the impact of repeated exposure to ethanol than adults, HMGB1 is induced by ethanol at all ages, which could contribute to epigenetic mechanisms altering microglial phenotypes that impact synapses and neurocircuits. Adolescent intermittent ethanol is known to induce anxiety and increase alcohol drinking through reversible epigenetic mechanisms that alter synaptic proteins [52, 151]. These findings are consistent with multiple studies finding neuroimmune activation promotes alcohol drinking which induces additional glial activation and epigenetic shifts in phenotypes across brain regions and cells (Figure 2).

Cholinergic neurons and hippocampal neuronal stem cells are two cell types presented as examples of how HMGB1-TLR proinflammatory signaling can directly alter neurocircuitry. AIE-induced loss of both ChAT+ neurons and hippocampal neurogenesis are prevented by indomethacin, an anti-inflammatory drug, and anti-cholinesterases, which increase acetylcholine and inhibit inflammation. Anti-inflammatory drugs are under investigation for treatment of AUD [63]. HMGB1-TLR4 signaling causes partial cholinergic neurons loss with remaining neurons shrunken due to induction of epigenetic silencing mechanisms. AIE-induced loss of ChAT+ neurons persists long into adulthood, likely for life, unless inhibited by anti-inflammatory or epigenetic drugs. This represents a phenotypic change in cholinergic phenotype. Although it is not clear, forebrain cholinergic-GABAergic neurons are common and lost ChAT+ neurons may remain GABAergic, altering target region circuitry. Cholinergic neurons respond to NGF, which is an important trophic factor reduced by AIE in target regions that could contribute to the reduced cholinergic transcriptome. This is consistent with proinflammatory-trophic transcription shifts in reducing cholinergic cellular phenotype. Interestingly, *in vivo* and *in vitro* reversal by anti-inflammatory, TLR4 antagonist or drugs that block epigenetic changes supports persistent proinflammatory signaling as maintaining epigenetic shifts in cholinergic phenotype. The reversal of epigenetic changes offers great promise for treatment of the chronic disease AUD. Changes in hippocampal neurogenesis similarly suggest proinflammatory increases and reduced BDNF trophic support alter the neurogenic niche, reducing adult hippocampal neurogenesis. Multiple cell types regulate the neurogenic niche and the proposed proinflammatory-induced shifts in phenotype are proposed for multiple cell types (Figure 2). Additional studies of how proinflammatory changes in cell transcriptomes and phenotypes contribute to progression to AUD across various brain regions will provide opportunities to develop improved treatments the have the promise of a cure through anti-inflammatory and epigenetic reversal of transcriptome shifting brain cell phenotypes.
